# Biological variability dominates and influences analytical variance in HPLC-ECD studies of the human plasma metabolome

**DOI:** 10.1186/1472-6890-7-9

**Published:** 2007-11-12

**Authors:** Yevgeniya I Shurubor, Wayne R Matson, Walter C Willett, Susan E Hankinson, Bruce S Kristal

**Affiliations:** 1 Department of Neurosurgery, Brigham and Women's Hospital, 221 Longwood Ave, LM322B, Boston, MA 02115, USA; 2 Burke Medical Research Institute, 785 Mamaroneck Ave., White Plains, NY 10605.^3 ^ESA, Inc., 22 Alpha Road, Chelmsford, MA 01824, USA; 3 Channing laboratory, Department of Medicine, Brigham and Women's Hospital, Harvard Medical School, Boston, MA 02115, USA; 4 Department of Nutrition, Harvard School of Public Health, 665 Huntington Ave, Boston, MA 02115, USA; 5 Department of Epidemiology, Harvard School of Public Health, 665 Huntington Ave, Boston, MA 02115, USA; 6 Department of Neuroscience, Cornell University Medical College, 1300 York Ave., NY, NY 10021, USA

## Abstract

**Background:**

Biomarker-based assessments of biological samples are widespread in clinical, pre-clinical, and epidemiological investigations. We previously developed serum metabolomic profiles assessed by HPLC-separations coupled with coulometric array detection that can accurately identify *ad libitum *fed and caloric-restricted rats. These profiles are being adapted for human epidemiology studies, given the importance of energy balance in human disease.

**Methods:**

Human plasma samples were biochemically analyzed using HPLC separations coupled with coulometric electrode array detection.

**Results:**

We identified these markers/metabolites in human plasma, and then used them to determine which human samples represent blinded duplicates with 100% accuracy (N = 30 of 30). At least 47 of 61 metabolites tested were sufficiently stable for use even after 48 hours of exposure to shipping conditions. Stability of some metabolites differed between individuals (N = 10 at 0, 24, and 48 hours), suggesting the influence of some biological factors on parameters normally considered as analytical.

**Conclusion:**

Overall analytical precision (mean median CV, ~9%) and total between-person variation (median CV, ~50–70%) appear well suited to enable use of metabolomics markers in human clinical trials and epidemiological studies, including studies of the effect of caloric intake and balance on long-term cancer risk.

## Background

After tobacco, over-nutrition is, arguably, the major cause of excess morbidity in developed countries, affecting a broad spectrum of diseases including cancer, cardio-/cerebrovascular disease, and type II diabetes. This association may be seen in both broad demographic groups, such as the American Cancer Society study group (900,000 U.S. adults)[1] and in more narrowly defined demographic groups, such as the Nurses' Health Study (NHS) group (122,000 U.S. female registered nurses) [2]. The difficulty of accurately assessing caloric intake and energy expenditure [3] has hampered studies relating to energy restriction, caloric balance, and caloric intake in both epidemiology and clinical nutrition. Several of the major hurdles in identifying biomarkers to address this and similar epidemiological problems are related to analytical (the lack of useful measurement standards) and methodological (the inability to distinguish individual physiology) issues [4-13]. Recent results have suggested the advantage of metabolomics approaches in clarifying these situations, at least for issues related to nutritional epidemiology[14,15].

Metabolomics technology [16,17] offers a promising new approach to identify biomarkers that characterize health and disease, including, as we have shown [18-21], caloric intake. The major advantage of metabolomic research in epidemiology and nutrition is that, at least in theory, metabolomics provides a snapshot view of a biological system and enables capture of information about both long- and short-term interactions of an organism and its environment, including nutrition. Thus, this approach provides us more complete information about the biochemical status or biochemical phenotype of organisms than many other possible approaches [22-25]. Position papers have remarked on the application of metabolomics to problems ranging from meat contamination to drug development and understanding mechanistic aspects of disease[26]. Within the realm of nutrition, metabolomics has been used to probe specific dietary constituents [27,28] and has been proposed as a key element in developing personalized medicine approaches [29-33] and in gaining insight into clinical and epidemiological questions [34,35].

Our metabolomics approach to clinical and epidemiological questions is distinct from, and complementary to, both direct targeted analysis (e.g., studying a few metabolites in a single pathway) and global profiling. Specifically, we focused initially on an animal model that displays the physiological benefits associated with nutritionally replete, lower-energy diets[18-21,36,37]. We propose that this analysis will enable us to address statistical concerns about the complexity of uninformed analysis of human datasets, harness the power of well-characterized animal models, and conserve finite biological samples from prospective epidemiologic cohorts. This work was conducted with a long-range focus on the use of epidemiological resources, tools, and approaches to develop individual risk predictors for humans and improved biomarkers for use in pre-clinical, clinical, and epidemiological studies.

We have previously completed proof of principle studies showing that we can identify serum metabolites that differ between AL-fed rats and rats undergoing CR[37], confirmed these findings in an independent cohort[18], and generated expert systems/trained algorithms that can objectively identify these groups [19,21]. We have further published a series of analytical reports related to the detailed methods of these studies, assessing the analytical variability and stability of the individual components in the plasma/sera metabolome[14,15,36,38,39]. Further characterization of these metabolomic serotypes in rats undergoing CR, including studies related to the duration and extent of restriction, is in progress (YS et al, in preparation). The next goal is to analyze the markers identified in these studies in humans. Before beginning these studies, however, we first need to confirm that our technological platform works with human plasma samples. We also must show that our overall platform, including collection and procurement methods, is robust within the constraints of prospective epidemiologic cohorts. In theory and in practice, the analytical variability of our measurements and the stability of the individual components of the plasma/sera metabolome could be assessed by simply determining the repeatability of the measurements of each marker, as we have described in detail in our previous papers [14,15].

As we move from studies of rat sera to studies of human plasma, however, many of the potential sources of error become both qualitatively different and quantitatively more complex. Here we address two of these issues: (i) our ability to measure these analytes reproducibly in banked human plasma and (ii) the need to assess the stability of these markers under different, realistic, and "worst case" shipping conditions. We note that, for the purposes of this report, we define this "worst case" in the context of the specific samples we expect to test in the future, which are drawn from the NHS. These samples are handled exactly as are the samples in the current report, and no sample is used that has been held >48 hours in shipping conditions.

## Methods

### Human plasma samples

Two sets of plasma samples that were collected into sodium heparin-containing tubes for use as analytical controls were examined in this study. Approximately 75% of the blood samples were drawn at least 8 hours after the last meal. In our study, all analysis of samples was conducted in a blinded fashion. Set 1 was comprised of duplicate (split) samples from 12 women who were participants in the NHS and triplicates (splits) of two pooled plasma samples. The latter samples were pools made up of multiple units of fresh frozen plasma obtained from a local hospital and used routinely to evaluate laboratory reproducibility. Set 2 consisted of three (split) samples from each of 10 individuals as well as duplicates of two pooled plasma samples as described above. These 10 adults were healthy men and women recruited locally who responded to a flyer requesting volunteers to provide blood samples for pilot studies. Of the three blood samples from each individual in Set 2, the first was processed (see below) immediately after acquisition, the second was stored as whole blood in a refrigerator for 24 hours, and the third was stored for 48 hours; these latter conditions mimicked typical overnight shipping conditions in many cohort studies. Processing consisted of centrifugation of whole blood samples at 1530 g for 20 min at 4°C, after which the plasma was removed and aliquots placed into cryotubes. These cryotubes containing plasma samples were frozen and maintained in the vapor phase of a liquid nitrogen freezer at <130°C. All nitrogen freezers were alarmed and monitored continuously. Samples were shipped from The Channing Laboratory to Burke Medical Research Institute by overnight courier on dry ice. Further details on this approach have been previously published[8].

IRB approval was obtained from Partners Human Research Committee and the IRB at Burke Medical Research Institute.

### HPLC-ECD analysis

Metabolite extraction, separation, detection, and identification were conducted as previously described. [14,15,36,38,40-45] (and see Additional File 1) Briefly, plasma samples were thawed to 0°C and distributed into new 1.5 mL microcentrifuge tubes (125 μl/tube). 500 μL of acetonitrile (An)/0.4% glacial acetic acid (HAc) at -20°C was added to each tube, after which the tubes were vortexed 20 sec and centrifuged for 15 min at 12000 g at -2°C. Supernatant in volumes of 500 μl was evaporated to dryness under vacuum in a CentriVap™ Concentrator (Labconco). The dry remains were dissolved in 100 μl of mobile phase A [see above refs, e.g. [38]] and placed in autosampler vials. Each set of samples was analyzed by HPLC within 2–3 days. The HPLC injection volume was 50 μl.

Chromatographic separation and electrochemical detection were performed using HPLC coupled with an electrochemical array detector (HPLC-ECD), as previously described[43,45]. The gradient and mobile phase reagents have also been previously described[43,45]. The reasons for the use of this protocol include integration of sample preparation and the mobile phases used. Notably the use of pentane sulfonic acid in mobile phase A solubilizes any protein fragments that may be extracted into the acetonitrile. The subsequent use of the B mobile phase containing virtually all organic solvents washes the column of any lipid materials which are extracted into the acetonitrile. This was discussed by in reports by Milbury [45]and Yao [46].

The gradient essentially displays an increase in hydrophobicity from that of ascorbate to that of tocopherol. Detection of metabolites was accomplished with a 16-channel coulometric array detector with potentials incremented in 60 mV steps (0–900 mV). All HPLC-ECD system functions were controlled by CoulArray software; biomarkers were identified and quantitated using CEAS-5.12 software. The metabolite concentration in individual human plasma samples was assessed and reported relative to that of metabolites in the "model" pool, in which the concentration of all markers was set at 100. The metabolic profile of the human pool studied in this report includes up to 66 markers, metabolites that represent a subset of the ~90 metabolites that we previously identified in sera of rats fed either AL or calorie restricted diets [14,18,19,21].

Most of the metabolites studied here are identified by virtue of their position in the array (retention time) and their relative reactivity across the array (dominant and subdominant channel). Examples of metabolites that can be assessed via Coularray-based technology include some amino acids (eg, tyrosine, tryptophan, cysteine, methionine), the majority of the tryptophan and tyrosine catabolic pathways, indoles, purines, antioxidants (eg ascorbate, tocopherol, glutathione, lipoate, dihydrolipoate) and redox damage products (eg, 8-OH-deoxyguanosine, glutathione disulfide).

### Mathematical/Statistical Analysis

Pearson correlation matrices were calculated in NCSS 97 with pairwise deletion for missing data. Means and errors, data simulations, and chi-squared analysis were conducted/determined in Microsoft Excel 2002. Paired t-tests were conducted in Statview. Principal components analysis was conducted using SIMCA P10.5 (Umetrics, Kinnelon, NJ). A single metabolite value, present at apparently 100-fold higher levels than any cognate metabolite in different samples, was excluded as an outlier.

## Results

Our previous analytical validation studies focused on identifying the sources of potential analytical error in our analyses, including methods of sample acquisition and preparation, handling, transportation, and storage, as well as the influence(s) of total series size, complexity of the organic matrix, and aspects of experimental design[8,15,47,48]. As noted above, we now continue these initial validation studies by examining both the reproducibility of the analytical platform when it is used to study human plasma and the stability of sample metabolites under simulated shipping conditions. Variability in sample acquisition (including variable stability under acquisition conditions) is fundamentally indistinguishable in our study from biological variability and therefore will be considered in a subsequent study.

### Analytical Reproducibility in Human Plasma

The reproducibility (precision) of metabolite measurements was addressed by blinded analysis of split samples. In the framework of a reproducibility study, aspects of the analytical platform such as the delivery of a sample to the analytical laboratory and the completion of sample analysis (e.g., sample processing), chromatographic separation and electrochemical detection, and peak identification and quantitation were considered.

To test our ability to analyze human plasma, we received 30 blinded samples, each of which was present in duplicate or triplicate (as a further blind, the laboratory was told that only duplicates were present). At the initial step, we looked for previously identified CR markers in the samples and identified 66 metabolites that clearly were present in serum/plasma from both species and were analytically suitable without further optimization.

Of the 23 remaining markers in our standard rat profile, 12 were not present in the plasma sample (expected, as some metabolites are only found in male rats), and 11 represented unclear assignments and were not studied further in this report.

**Figure 1:**
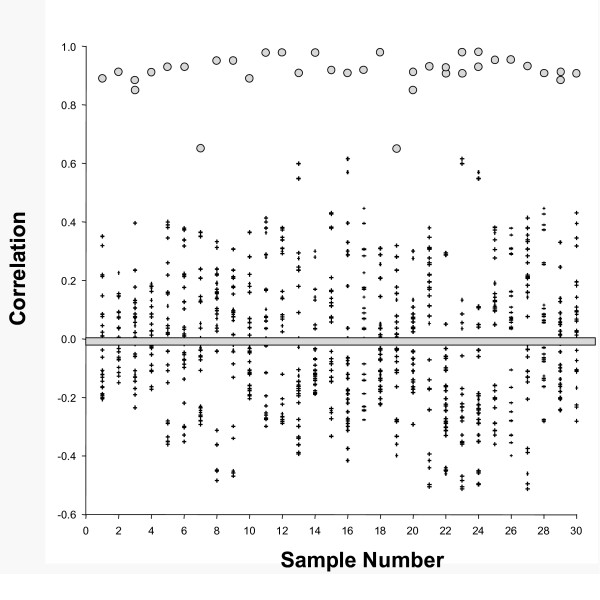
**Correlation analysis of human plasma based on 66 metabolites**. Pearson correlations of each of 30 samples with each of the other 29 samples are presented graphically. Each datapoint represents the mean correlation for the 66 metabolites between the specific sample and each of the other 29 samples. Filled circles show the correlation of a sample with its correct duplicate or triplicate. Small pluses show the correlation of a sample with unmatched samples. A heavy bar has been placed at zero for visual reference.

Using these 66 metabolites, we readily identified all 30 of the blinded duplicates and triplicates. This demonstration of *analytical *self-similarity represents the first stage in validating the analytical platform for future use in human studies. In all 30 cases, cognate duplicates/triplicates were immediately apparent (Figure 1). Quantitative data show that, for all comparisons, the highest correlations were with the cognate split. Although the dataset is small, these findings also begin to address biological variability. Specifically, the mean correlation between two non-matched samples was 0.004 ± 0.20 (mean ± S.D.) and the median correlation was -0.007. These numbers suggest a relatively low correlation between samples derived from different individuals. This analysis does not address, however, the relative variability between multiple samples from a given individual as compared with samples derived from different individuals (see below and work in progress).

Having demonstrated high precision on a per sample basis, we then evaluated precision at the individual metabolite level. Fourteen duplicate samples (12 of which were derived from individuals and 2 of which were pooled plasma samples) were scored for the 66 markers (924 total metabolites). The resulting data were then used to estimate the analytical accuracy that could be obtained from the analysis of banked human plasma (Table 1). The data were analyzed before and after manually reconfirming any peak with a coefficient of variation (CV) of >40% (48 pairs, 96 total metabolites); this procedure is referred to as "polishing" in Table 1, and was done specifically to determine if the analytical error originated before or during the HPLC analysis or during peak matching and quantitation.

**Table 1:** Analytical Parameters

	Before polishing data	After polishing data
Range, median CVs for 66 variables	2–54%	2–36%
Range, mean CVs for 66 variables	2–58%	3–39%
**Median CV for all 66 variables**	**12%**	**12%**
Mean CV for all 66 variables	19%	17%
**By 13 Pairs, overall median CV**	**9%**	**9%**

"Before polishing data", primary (raw) analysis; "After polishing data", final analysis after manual re-inspection (see text). The most critical data are the median coefficients of variation (CV); our median CV was ~10%, well within the levels necessary to conduct the proposed study. We also note that upon re-examination of the dataset for quality control purposes, 1% (11 peaks) was left unscored due to analytical concerns, and 3% (25 peaks) had scores that differed by >10% from the original call. These data suggest that, even in the absence of any optimization, we can consistently measure >96% of all metabolites with a median ± 10% precision. For the last line "By 13 pairs", we first determined the median CV for all 66 variables in each of the pairs, and then determined the mean/median of these values. By definition, overall mean CVs are unchanged by this difference in calculation procedure, and are not shown.

Fewer than 4% of the data points were found to have peak matching and quantitation errors of >10%, see legend to Table 1). The comparisons were made across the dataset and between the pairs. Both mean and median values of the measurements are reported to stress that analysis of the majority of analytes had very good quantitative reproducibility. This finding supports the contention that most of the cross-species markers can be measured with sufficient analytical accuracy for use in additional studies. Within subject quantitative reproducibility of the measurement of metabolite concentrations in human plasma was comparable with data obtained for rat sera (rat sera: mean CV of ~12%, median CV of ~7%; human plasma: mean CV of 17–19%, median CV of ~12%, see Table 1) [14,15].

### Sample Stability under "Worst Case" Shipping Conditions

In contrast to analysis of samples in laboratory-based research, analysis of samples in population-based studies is complicated by the need to transfer specimens from the field to a central location, where they are then processed and stored. In epidemiology studies, this requirement often means that whole blood samples cannot be frozen prior to arrival at the central location and subsequent processing. In practice, this constraint imparts a delay, generally approximately 24 hours but potentially as great as 48 hours, between the time of collection and the time of analysis or freezing for long-term storage. It is therefore essential to confirm that these delays do not destroy or severely degrade the analytes of interest. We used a testing procedure developed within the NHS to address this issue.

**Figure 2:**
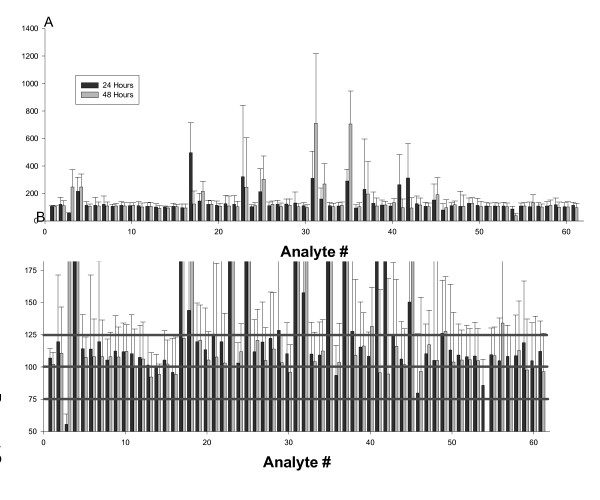
**Stability study of 61 metabolites**. 34 plasma samples held under shipping conditions for 0, 24, or 48 hours were run and analyzed using standard protocols. Data are presented as mean +/- SD from the 0 time point. Each analysis is based on data from 10 samples analyzed at 3 time points each. Left panel shows a "zoomed-out" view to give an overview of the structure of the data; right panel shows a "zoomed-in" view to give a better sense of the fine structure of the data. The pink lines in the right figure mark +/- 25%.

In the NHS, the samples are collected from locations all over the country, thus variation in time of collection, time exposed to light prior to packaging, and time devoted to shipping are impossible to control. We therefore examined samples that had been allowed to sit for 0, 24, or 48 hours under simulated shipping conditions prior to processing. Of the 61 metabolites that we tested (three were eliminated after displaying relatively poor reproducibility in the above study; two were not clearly identified in this follow-up study), 46 (~75%) showed variation consistent with normal analytical variation over this time frame (Figure 2).

**Figure 3:**
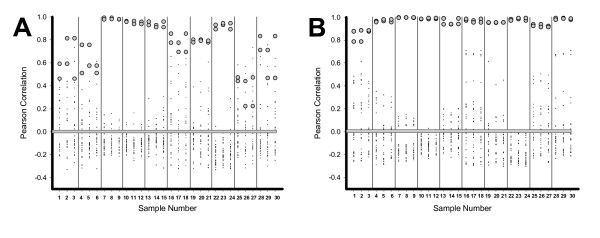
**Correlation analysis of human plasma based on 66 or 46 metabolites**. Pearson correlations of each of 3 sets of 10 samples with each of the other 29 samples are presented graphically. Triplicates include one sample that was not incubated prior to freezing, one incubated for 24 hours, and one incubated for 48 hours. Each datapoint represents the mean correlation for the 61 (Panel A) or 46 metabolites between the specific sample and each of the other 29 samples. Filled circles show the correlation of a sample with its correct triplicate. Small pluses show the correlation of a sample with unmatched samples. A heavy bar has been placed at zero for visual reference. Thin vertical lines are used to visually highlight the groups.

The use of correlation-based approaches (as above) enabled us to assign 29 of 34 samples to the individual from whom the sample was derived (10 triplets [0, 24, 48 hours] – Figure 3, Panel A, 2 duplicates as internal controls – data not shown). Specifically, only four samples could not be definitively assigned to the correct individual, and only one was assigned incorrectly (2 pairs of duplicate pools were assigned correctly; 7 triplets from individuals were assigned correctly; for 2 triplets, 2 of 3 samples were matched correctly, whereas 1 was not assigned; for 1 triplet, 2 samples were not assigned [marked as possible match] and 1 was assigned incorrectly). Thus, even under the worst possible test conditions (i.e., all unstable metabolites included, samples outside normal temporal bounds, no algorithm optimization), we can still identify the origin of 85% of all samples with only 3% absolute error. When only the 46 analytically strong peaks are used, the individual origin of each sample is apparent with 100% accuracy (Figure 3, Panel B).

### Biological variability of markers

We next addressed the inherent biological variability of the human metabolome. Initial studies on biological variability were conducted in two ways: by comparison of the markers as a population, and by comparisons of individual markers. In both cases, the concentration of each marker was normalized to its corresponding concentration in a control pool; i.e., we are discussing relative rather than absolute concentrations of the metabolites in the profile. When the metabolites are considered as a population and scored as percents of a standardized pool, test set 1 and set 2 (control group) had essentially equivalent levels of metabolites (Table 2, top line, means). Overall, however, metabolite levels varied more widely in the second population than in the first. Specifically, the levels of the metabolites measured showed both greater quantitative variability (Table 2, top line, S.D.s) and larger ranges [mean level of metabolites varied between individuals from ~80–140% and ~65–130% (~1.8–2-fold) in set 1 and from 45–150% (~3.3-fold) in set 2]. This increased variability may relate, at least in part, to the inclusion of plasma from both sexes in cohort 2. Comparison of means and medians suggest that this difference was caused by the presence of more extreme values in the second group rather than a shift in the central tendency.

**Table 2:** Descriptive statistic of total biomarker levels in human plasma

	**Metabolite measurements**			
**Statistics**	1^st ^set of data (n = 14)*	2^nd ^set of data (n = 10)**		
	
	**Duplicates &Triplicates**	**Control**	**24-hours**	**48-hours**

**Mean ± SD**	94.8± 52.5	85.1 ± 75.1	96.2 ± 83.4	97.6 ± 77.6
**Mean median**	82.3	60.1	67.7	71.8
**Mean CV**	57.1	82.7	83.8	76.4
**Median CV**	50.2	70.6	70.7	66.9

* 66 metabolites

** 61 metabolites

Mean ± SD: Each metabolite is scored relative to the standard pool, then each individual sample is assigned one value (the mean of its metabolites averaged across the duplicates and triplicates) and these means are then expressed as mean ± SD

Mean Median: Average of the medians of the 66/61 variables across the population

Mean CV: Average of the CVs of the 66/61 variables across the population

Median CV: Median of the CVs of the 66/61 variables across the population

**Figure 4:**
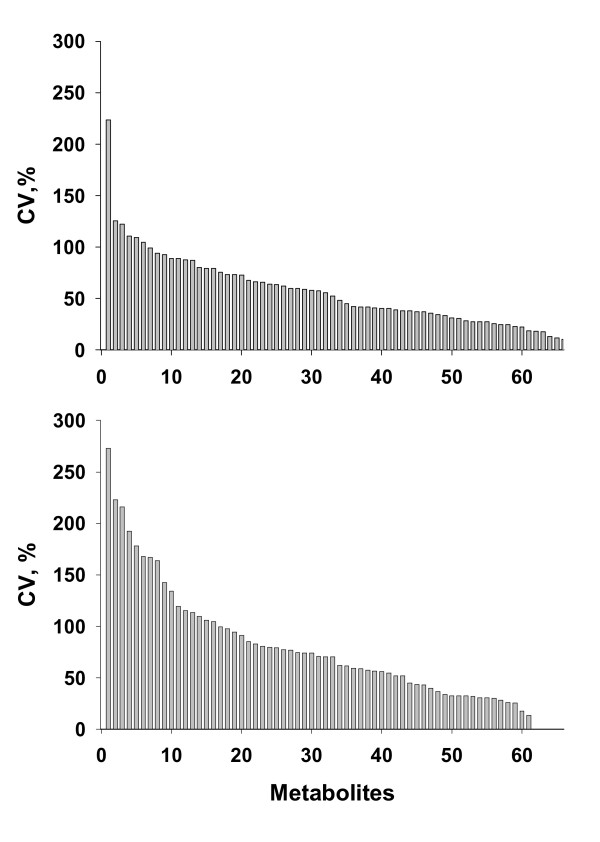
**Biological variability of markers**. Biological variability of metabolite concentrations assessed in set 1 (A, 14 samples, 66 metabolites) and set 2 (B, 10 samples, 61 metabolites, 0 time point) of human plasma samples. All data are presented as CV (%), and sorted from largest to smallest CVs to simplify comparisons.

Individual biomarkers also showed wide ranges in their biological variability. As shown in Figure 4, less than 8% of our markers had inter-person CVs of <20%, approximately equivalent to the percentage that had CVs exceeding 100%. Variability, here defined at the level of individual metabolites, was again slightly greater in the second population, similar to that seen at the level of the markers as a population. Expansion of our studies to broader populations may further refine these estimated distributions.

**Figure 5:**
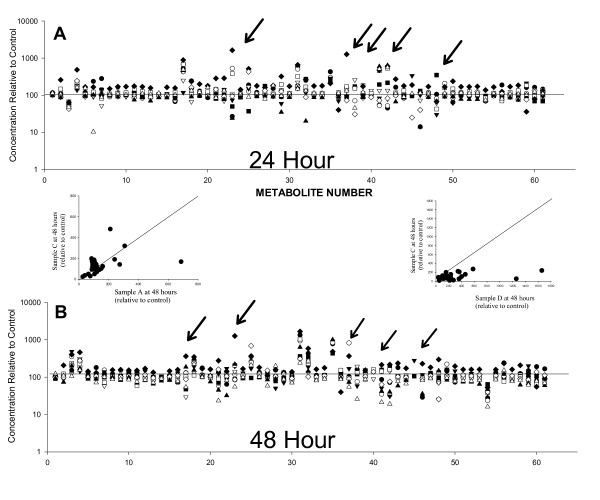
**Biomarkers display metabolite- and individual-specific degradation**. Panels A and B show the concentrations of the metabolites studied, at 24 (Panel A) and 48 hours (Panel B) under shipping conditions. Data are expressed as percentage of the original (Time 0) concentration. Arrows highlight some of the particularly variable metabolites. Note the log scale to emphasize fold-change. The left insert shows an example of two 48 hour samples in which individual changes are metabolite specific; the right insert shows a comparison in which almost all metabolites are more sensitive to time-associated changes in one individual than another. The diagonal lines have been added to emphasize the point at which metabolites undergoing equivalent degradation in both individuals would be located. Data from the study of plasma samples from 10 individuals are shown.

**Figure 6:**
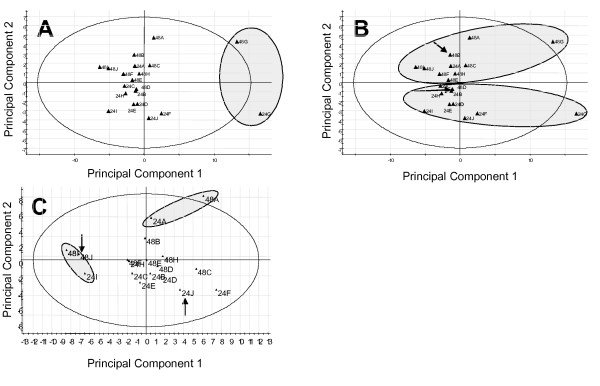
**Principal Components analysis suggests that multiple individualized degradation patterns exist**. Panels A and B show a principal components analysis (components 1 and 2) of the entire dataset (61 variables, 10 individuals, 24 and 48 hour time points, expressed relative to t = 0 control sample – ie the data shown in Figure 5). A shaded oval has been added to panel A to show that metabolites from one individual are much more generally sensitive to degradation than those of the others. The shaded ovals in panel B show that this informatics approach readily recognizes the 24 and 48 hour time groups, with the two "exceptions" denoted with arrows. Panel C shows a different principal components analysis (after removal of 24 and 48G from panel A), with the 24 and 48 hour time points from two individuals identified with colored ovals; note that the long axis of the ovals are orthogonal. A similar but visually more difficult relationship to interpret is seen with sample A and F (sample 48F sits just left of the origin at [^~^-2,0]) Arrows denote two samples in which an individual's samples did not group.

It is reasonable to expect that metabolites from different individuals/samples will show essentially equivalent changes during storage and processing (ie, analytical parameters for a given variable are assumed to be independent of the source). This result was observed for most markers when they were compared between most individuals in our data set (Figure 5). Samples from one individual, however, showed elevated degradation of multiple markers (Figure 5, right insert, Figure 6, Panel A); furthermore, increased concentrations of several metabolites were observed relative to the control in some samples from this individual, whereas decreased concentrations of these metabolites were observed in others (Figure 5). In some cases, changes in metabolites were biphasic between 0, 24, and 48 hours, suggesting the presence of either competing or sequential reactions. Consistent with this possibility, considerable inter-individual (Figure 6) and inter-metabolite (Figure 5) differences were observed at both 24 and 48 hours time points. Nonetheless, principal components analysis of the metabolic profiles readily distinguished 24 and 48 hour samples with 90% accuracy (Figure 6, panel B [note: 0 and 24 hour samples could not be so separated]). These differences in component 2 were statistically significant (p = 0.0001 by paired t-test). Furthermore, note the essentially orthogonal distinction between the effects of transport conditions on samples A and I (Figure 6, Panel C, see legend for note on individual F), and the clear biphasic response of individual J (Figure 6, panel C, arrows).

To assess the statistical validity of this observation of apparent inter-personal differences in compound stability, we compared all metabolite changes by paired t-test (10 triplets means 45 paired t-test comparisons [10*9/2] at each time point, 24 and 48 hours). Twenty of 45 comparisons had p < 0.05 at 24 hours, and 22 of 45 had p < 0.05 at 24 hours. To assess the likelihood of this result occurring by chance, we modeled changes in metabolite levels assuming that all changes were random. Of 100 comparisons, 6 had p values < 0.05 (consistent with expected results from probability alone). Chi-squared analysis of the comparison resulted in p values of < 10^-10^.

## Discussion

The central finding of this paper is that the metabolites that we have previously used to distinguish caloric intake in rats can be analyzed in human plasma with good analytical precision (median CV of 9–12%) and have high inter-sample variability (median CV of 50–70%). This combination, similar to results obtained from rat studies, [14] suggests that these markers are analytically suited for use in studies of the serum metabolome in human epidemiologic cohorts and multi-center clinical trials. [Note: Analytical CVs are slightly higher in the human samples. This slightly increased variability in human plasma versus rat sera might relate to the procedure of human plasma preparation, which includes the addition of anticoagulants to the blood. Analysis of human plasma as compared with rat sera was associated with a more rapid contamination of guard and analytical columns and greater wearing of the electrodes, suggesting that even the acetonitrile purified sample retained some contaminants, which might also degrade performance.]

Two important caveats follow from our experimental design: (i) the estimate of total inter-sample variability includes both the analytical variability and the biological variability, although the study's overall analytical precision suggests that the variability is primarily biological in origin; and (ii) we cannot distinguish the components of biological variability that derive from sample-to-sample within-person variability versus long-term between-person variability – the latter of which is critical for our planned investigations. Work on this latter question, the relative biological variability between different people as compared to the variability within a temporal series of samples from a given person, is the next logical step.

In general, biomarker validation studies require demonstrating validity in three broadly defined stages, in which the following concerns are addressed: (i) analytical issues [8,47,48]; (ii) inter- vs intra-personal biological variation; and (iii) utility (ie, the correspondence of a certain biomarker profile with a phenotype of interest) [4,7,49-57].

A critical inherent assumption in most or all biomarker studies is that, from an analytical/mathematical standpoint, stage (i) must precede stage (ii) and that stage (i) is essentially independent of stage (ii). In part, this logical construction simply states that we must be able to measure the concentration of an analyte, and understand the limits of that measurement, before we can usefully examine differences in that analyte between two or more conditions of interest. The above logical construction further implies that our ability to measure a given analyte and the basic analytical properties of that analyte are expected to be unaffected by its source – that is, the person from whom the sample is derived (e.g., the accuracy of measuring the sodium concentration in a blood sample is expected to be equivalent in identically-treated samples from different people).

Our results appear to provide empirical evidence supporting a noteworthy exception to this logical, but ultimately theoretical argument. These data, and the interpretation of these data, are dependent on the extent to which sample handling in our experiment was sufficiently controlled to enable other influences to be excluded. In support of the idea that we met these conditions, the differences observed are primarily in the 48 hour samples, whereas we would expect random distribution for most possible analytical problems (e.g., sample handling). Further support for our ability to generally fulfill the goal of appropriate sample handling is provided by evidence of high correlations between the levels of corresponding metabolites in paired samples (See Figures 1 and 3). Nonetheless, although we tried to treat all samples equally, it is impossible to exclude the possibility that there was some unrecognized difference in handling that contributed to the observed individual degradation patterns. Given this caveat, however, our results provide evidence that, especially at 48 hours, but even, to a lesser extent, at 24 hours, individual differences in bio-or chemo-transformation of metabolites (i.e., differences in metabolite stability) exist at a measurable level. At 48 hours, these differences are sufficient to enable ready classification by time in simulated shipping conditions, suggesting that avoiding 48 hour delays in initial sample processing is strongly desired. Because relatively fast processing is not always possible, the development of new methods for recognizing excessive transformation/degradation of metabolites would be helpful, allowing "for cause" exclusion of outlying samples if necessary.

Attempts to distinguish the existence of different groups or classes of individuals with respect to their metabolomic transformation appeared suggestive, but were statistically borderline with respect to overfit diagnostics and are not shown. We have no direct evidence as to the mechanism of metabolite transformation, and can only suggest that the interplay between genetics and environment and between enzymatic and non-enzymatic mechanisms might be involved in the variability of biomarker degradation. These data suggest that, for the case of plasma metabolomics analysis relevant for epidemiological studies, the general assumption that biological and analytical variation are independent must be viewed with caution, as there appear to be some individual-specific, metabolite-specific interactions. For studies such as ours, these concerns, if they occur in significant numbers, would show up as loss of signal and increase in noise, with a consequent reduction in the signal:noise ratio. From what we have seen, this issue is not a major concern in our study. The recognition and/or understanding of such changes might, however, be particularly important if one attempts to bring a quasi-mechanistic systems biology approach to deriving models for study in epidemiological cohorts. Consider, for example, a disease hypothesized to result from a failure of homeostatic feedback among the compartments of the genome, transcriptome, proteome, and metabolome. A metabolomic model of this disease could be built based on animal studies in which sample handling is (or, at least, can be) rigorously defined, but would be difficult to address in humans due to both biological and analytical noise. Understanding analytical noise is thus one step toward enabling study of mechanistic hypotheses in humans.

## Conclusion

In conclusion, it is worth recalling that the development and validation of biomarkers of nutritional status for use in human studies has a long history. Despite this history, relatively few useful markers – other than direct intake markers such as carotenoids in blood, double-labeled water, and urinary nitrogen – have been identified and are in widespread use for dietary status assessment [34,58-60]. Attempts to identify biomarkers of direct dietary intake have been limited by many factors, including both analytical and biological issues[34,61-68]. In this report, we present evidence that biomarker profiles reflecting two extremes of caloric intake in rodents can be adapted for use in humans. This profile is analytically stable at the level of both population and individual markers, with median analytical CVs < 20% of median biological CVs, even under the worst case shipping conditions and the inclusion of markers with lower analytical quality (defined here as stability). The surprising finding was that the stability of some markers clearly varied between individuals. This finding suggests that sources of variation normally considered as analytical can be influenced by biological parameters.

## Abbreviations

CR: Calorie-restricted;

HPLC-ECD: HPLC coupled with an electrochemical array detector;

NHS: Nurses' Health Study.

## Competing interests

Dr. Matson was a founder and former director of ESA, Inc, which makes the analytical equipment used in this study, but he no longer has a financial interest in the company. Both Drs. Kristal and Matson consult for and have equity interest in Metabolon, a metabolomics company, but the work appears unrelated to Metabolon.

Dr. Kristal has equity and potential royalties from licensed intellectual property to Metabolon (through Weill Medical College of Cornell University), but this patent is also not directly related to the work presented here.

Drs Kristal and Matson were joint inventors on a patent on biomarkers of caloric restriction, which is unlicensed. Dr. Kristal's interest is again overseen by Weill Medical College of Cornell University (and held jointly with University of Texas Health Science Center at San Antonio), Dr. Matson's interest is wholly owned by ESA, inc.

## Authors' contributions

YIS contributed to the analytical design of this work, conducted and analyzed all HPLC analysis, and drafted the manuscript, WRM contributed to the overall/general conception and design of the HPLC analysis, WCW and SEH contributed to the conception and design of the human sample studies in this work, BSK conducted the statistical and informatics analysis and drafted the manuscript. All authors contributed to the overall design of the larger project of which this work is a part, and all authors were involved in editing and approving the manuscript

## Pre-publication history

The pre-publication history for this paper can be accessed here:



## References

[B1] Calle EE, Rodriguez C, Walker-Thurmond K, Thun MJ (2003). Overweight, obesity, and mortality from cancer in a prospectively studied cohort of U.S. adults. N Engl J Med.

[B2] Willett WC, Dietz WH, Colditz GA (1999). Guidelines for healthy weight. N Engl J Med.

[B3] Willett WC (1998). Nutritional Epidemiology.

[B4] Potischman N (2003). Biologic and methodologic issues for nutritional biomarkers. J Nutr.

[B5] Potischman N, Freudenheim JL (2003). Biomarkers of nutritional exposure and nutritional status: an overview. J Nutr.

[B6] Marshall JR (2003). Methodologic and statistical considerations regarding use of biomarkers of nutritional exposure in epidemiology. J Nutr.

[B7] Blanck HM, Bowman BA, Cooper GR, Myers GL, Miller DT (2003). Laboratory issues: use of nutritional biomarkers. J Nutr.

[B8] Hankinson SE, London SJ, Chute CG, Barbieri RL, Jones L, Kaplan LA, Sacks FM, Stampfer MJ (1989). Effect of transport conditions on the stability of biochemical markers in blood. Clin Chem.

[B9] Tuxen MK, Soletormos G, Petersen PH, Schioler V, Dombernowsky P (1999). Assessment of biological variation and analytical imprecision of CA 125, CEA, and TPA in relation to monitoring of ovarian cancer. Gynecol Oncol.

[B10] Petersen PH, Fraser CG, Jorgensen L, Brandslund I, Stahl M, Gowans E, Libeer JC, Ricos C (2002). Combination of analytical quality specifications based on biological within- and between-subject variation. Ann Clin Biochem.

[B11] Bingham SA, Gill C, Welch A, Cassidy A, Runswick SA, Oakes S, Lubin R, Thurnham DI, Key TJ, Roe L (1997). Validation of dietary assessment methods in the UK arm of EPIC using weighed records, and 24-hour urinary nitrogen and potassium and serum vitamin C and carotenoids as biomarkers. Int J Epidemiol.

[B12] Saracci R (1997). Comparing measurements of biomarkers with other measurements of exposure. IARC Sci Publ.

[B13] Key T, Oakes S, Davey G, Moore J, Edmond LM, McLoone UJ, Thurnham DI (1996). Stability of vitamins A, C, and E, carotenoids, lipids, and testosterone in whole blood stored at 4 degrees C for 6 and 24 hours before separation of serum and plasma. Cancer Epidemiol Biomarkers Prev.

[B14] Shurubor YI, Paolucci U, Krasnikov BF, Matson WR, Kristal BS (2005). Analytical Precision, Biological Variation, and Mathematical Normalization in High Data Density Metabolomics. Metabolomics.

[B15] Shurubor Y, Matson WR, Martin RJ, Kristal BS (2005). Relative Contribution of Specific Sources of Systematic Errors and Analytical Imprecision to Metabolite Analysis by HPLC-ECD. Metabolomics.

[B16] Vaidyanathan S, Harrigan GG, Goodacre R (2005). Metabolome Analysis: Strategies for Systems Biology.

[B17] Harrigan GG, Goodacre R (2003). Metabolic Profiling: Its Role in Biomarker Discovery and Gene Function Analysis.

[B18] Shi H, Vigneau-Callahan KE, Shestopalov AI, Milbury PE, Matson WR, Kristal BS (2002). Characterization of diet-dependent metabolic serotypes: Primary validation of male and female serotypes in independent cohorts of rats. J Nutr.

[B19] Shi H, Paolucci U, Vigneau-Callahan KE, Milbury PE, Matson WR, Kristal BS (2004). Development of Biomarkers based on Diet-Dependent Metabolic Serotypes: Practical Issues in Development of Expert System-Based Classification Models in Metabolomic Studies. OMICS J Integr Biol.

[B20] Paolucci U, Vigneau-Callahan KE, Shi H, Matson WR, Kristal BS (2004). Development of Biomarkers Based on Diet-Dependent Metabolic Serotypes: Concerns and Approaches for Cohort and Gender Issues in Serum Metabolome Studies. OMICS J Integr Biol.

[B21] Paolucci U, Vigneau-Callahan KE, Shi H, Matson WR, Kristal BS (2004). Development of Biomarkers Based on Diet-Dependent Metabolic Serotypes: Characteristics of Component-based Models of Metabolic Serotype. OMICS J Integr Biol.

[B22] Fiehn O (2002). Metabolomics – the link between genotypes and phenotypes. Plant Mol Biol.

[B23] Bino RJ, Hall RD, Fiehn O, Kopka J, Saito K, Draper J, Nikolau BJ, Mendes P, Roessner-Tunali U, Beale MH (2004). Potential of metabolomics as a functional genomics tool. Trends Plant Sci.

[B24] Fiehn O, Kopka J, Dormann P, Altmann T, Trethewey RN, Willmitzer L (2000). Metabolite profiling for plant functional genomics. Nat Biotechnol.

[B25] Weckwerth W, Loureiro ME, Wenzel K, Fiehn O (2004). Differential metabolic networks unravel the effects of silent plant phenotypes. Proc Natl Acad Sci USA.

[B26] Goodacre R, Vaidyanathan S, Dunn WB, Harrigan GG, Kell DB (2004). Metabolomics by numbers: acquiring and understanding global metabolite data. Trends Biotechnol.

[B27] Solanky KS, Bailey NJ, Beckwith-Hall BM, Bingham S, Davis A, Holmes E, Nicholson JK, Cassidy A (2005). Biofluid 1H NMR-based metabonomic techniques in nutrition research – metabolic effects of dietary isoflavones in humans. J Nutr Biochem.

[B28] Lamers RJ, DeGroot J, Spies-Faber EJ, Jellema RH, Kraus VB, Verzijl N, TeKoppele JM, Spijksma GK, Vogels JT, van der GJ (2003). Identification of disease- and nutrient-related metabolic fingerprints in osteoarthritic Guinea pigs. J Nutr.

[B29] German JB, Roberts MA, Watkins SM (2003). Genomics and metabolomics as markers for the interaction of diet and health: lessons from lipids. J Nutr.

[B30] Watkins SM, German JB (2002). Toward the implementation of metabolomic assessments of human health and nutrition. Curr Opin Biotechnol.

[B31] German JB, Roberts MA, Fay L, Watkins SM (2002). Metabolomics and individual metabolic assessment: the next great challenge for nutrition. J Nutr.

[B32] Watkins SM, German JB (2002). Metabolomics and biochemical profiling in drug discovery and development. Curr Opin Mol Ther.

[B33] Noguchi Y, Sakai R, Kimura T (2003). Metabolomics and its potential for assessment of adequacy and safety of amino acid intake. J Nutr.

[B34] Prentice RL, Willett WC, Greenwald P, Alberts D, Bernstein L, Boyd NF, Byers T, Clinton SK, Fraser G, Freedman L (2004). Nutrition and physical activity and chronic disease prevention: research strategies and recommendations. J Natl Cancer Inst.

[B35] Milner JA (2003). Incorporating basic nutrition science into health interventions for cancer prevention. J Nutr.

[B36] Vigneau-Callahan KE, Shestopalov AI, Milbury PE, Matson WR, Kristal BS (2001). Characterization of Diet-Dependent Metabolic Serotypes: Analytical and Biological Variability Issues in Rats. J Nutr.

[B37] Shi H, Vigneau-Callahan KE, Shestopalov AI, Milbury PE, Matson WR, Kristal BS (2002). Characterization of diet-dependent metabolic serotypes: Proof of principle in female and male rats. J Nutr.

[B38] Shi H, Vigneau-Callahan KE, Matson WR, Kristal BS (2002). Attention to relative response across sequential electrodes improves quantitation of coulometric array. Anal Biochem.

[B39] Kristal BS, Shurubor Y, Paolucci U, Matson WR, Harrigan G, Goodacre R, Vaidyanathan S (2005). Methodological issues and experimental design considerations to facilitate development of robust, metabolic profile-based classification. Metabolic Profiling: Its Role in Drug Discovery and Integration with Genomics and Proteomics.

[B40] Matson WR, Gamache PH, Beal MF, Bird ED (1987). EC array sensor concepts and data. Life Sci.

[B41] Matson WR, Bouckoms A, Svendson C, Beal MF, Bird ED (1990). Generating and controlling multiparameter databases for biochemical correlates of disorders. Basic, clinical and therapeutic aspects of Alzheimer's and Parkinson's diseases.

[B42] Matson WR, Langials P, Volicer L, Gamache PH, Bird ED, Mark KA (1984). n-electrode three dimensional liquid chromatography with electrochemical detection for determination of neurotransmitters. Clinical Chem.

[B43] Kristal BS, Vigneau-Callahan KE, Matson WR (1998). Simultaneous analysis of the majority of low-molecular-weight, redox-active compounds from mitochondria. Anal Biochem.

[B44] Kristal BS, Vigneau-Callahan KE, Matson WR (2002). Simultaneous analysis of multiple redox-active metabolites from biological matrices. Methods in Molecular biology.

[B45] Milbury PE (1997). CEAS generation of large multiparameter databases for determining categorical process involvement of biomolecules. Coulometric Array Detectors for HPLC.

[B46] Yao JK, Cheng P (2004). Determination of multiple redox-active compounds by high-performance liquid chromatography with coulometric multi-electrode array system. J Chromatogr B Analyt Technol Biomed Life Sci.

[B47] Clark S, Youngman LD, Chukwurah B, Palmer A, Parish S, Peto R, Collins R (2004). Effect of temperature and light on the stability of fat-soluble vitamins in whole blood over several days: implications for epidemiological studies. Int J Epidemiol.

[B48] Clark S, Youngman LD, Palmer A, Parish S, Peto R, Collins R (2003). Stability of plasma analytes after delayed separation of whole blood: implications for epidemiological studies. Int J Epidemiol.

[B49] Fraser CG (2004). Test result variation and the quality of evidence-based clinical guidelines. Clin Chim Acta.

[B50] Ricos C, Iglesias N, Garcia-Lario JV, Simon M, Cava F, Hernandez A, Perich C, Minchinela J, Alvarez V, Domenech MV, Jiménez CV, Biosca C, Tena R (2007). Within-subject biological variation in disease: collated data and clinical consequences. Ann Clin Biochem.

[B51] Dalle-Donne I, Rossi R, Colombo R, Giustarini D, Milzani A (2006). Biomarkers of oxidative damage in human disease. Clin Chem.

[B52] Ricos C, Domenech MV, Perich C (2004). Analytical quality specifications for common reference intervals. Clin Chem Lab Med.

[B53] Aitio A, Apostoli P (1995). Quality assurance in biomarker measurement. Toxicol Lett.

[B54] Baker M (2005). In biomarkers we trust?. Nat Biotechnol.

[B55] Livingstone MB, Black AE (2003). Markers of the validity of reported energy intake. J Nutr.

[B56] Wild CP, Andersson C, O'Brien NM, Wilson L, Woods JA (2001). A critical evaluation of the application of biomarkers in epidemiological studies on diet and health. Br J Nutr.

[B57] Crews H, Alink G, Andersen R, Braesco V, Holst B, Maiani G, Ovesen L, Scotter M, Solfrizzo M, van den BR (2001). A critical assessment of some biomarker approaches linked with dietary intake. Br J Nutr.

[B58] McKeown NM, Day NE, Welch AA, Runswick SA, Luben RN, Mulligan AA, McTaggart A, Bingham SA (2001). Use of biological markers to validate self-reported dietary intake in a random sample of the European Prospective Investigation into Cancer United Kingdom Norfolk cohort. Am J Clin Nutr.

[B59] Trabulsi J, Troiano RP, Subar AF, Sharbaugh C, Kipnis V, Schatzkin A, Schoeller DA (2003). Precision of the doubly labeled water method in a large-scale application: evaluation of a streamlined-dosing protocol in the Observing Protein and Energy Nutrition (OPEN) study. Eur J Clin Nutr.

[B60] Okubo H, Sasaki S, Rafamantanantsoa HH, Ishikawa-Takata K, Okazaki H, Tabata I (2007). Validation of self-reported energy intake by a self-administered diet history questionnaire using the doubly labeled water method in 140 Japanese adults. Eur J Clin Nutr.

[B61] Subar AF, Kipnis V, Troiano RP, Midthune D, Schoeller DA, Bingham S, Sharbaugh CO, Trabulsi J, Runswick S, Ballard-Barbash R, Sunshine J, Schatzkin A (2003). Using intake biomarkers to evaluate the extent of dietary misreporting in a large sample of adults: the OPEN study. Am J Epidemiol.

[B62] Marshall JR (2003). Methodologic and statistical considerations regarding use of biomarkers of nutritional exposure in epidemiology. J Nutr.

[B63] Tooze JA, Schoeller DA, Subar AF, Kipnis V, Schatzkin A, Troiano RP (2007). Total daily energy expenditure among middle-aged men and women: the OPEN Study. Am J Clin Nutr.

[B64] Midthune D, Kipnis V, Freedman LS, Carroll RJ (2007). Binary Regression in Truncated Samples, with Application to Comparing Dietary Instruments in a Large Prospective Study. Biometrics.

[B65] Thompson FE, Kipnis V, Midthune D, Freedman LS, Carroll RJ, Subar AF, Brown CC, Butcher MS, Mouw T, Leitzmann M, Schatzkin A (2007). Performance of a food-frequency questionnaire in the US NIH-AARP (National Institutes of Health-American Association of Retired Persons) Diet and Health Study. Public Health Nutr.

[B66] Thompson FE, Midthune D, Subar AF, Kipnis V, Kahle LL, Schatzkin A (2007). Development and evaluation of a short instrument to estimate usual dietary intake of percentage energy from fat. J Am Diet Assoc.

[B67] Lissner L, Troiano RP, Midthune D, Heitmann BL, Kipnis V, Subar AF, Potischman N (2007). OPEN about obesity: recovery biomarkers, dietary reporting errors and BMI. Int J Obes (Lond).

[B68] Dodd KW, Guenther PM, Freedman LS, Subar AF, Kipnis V, Midthune D, Tooze JA, Krebs-Smith SM (2006). Statistical methods for estimating usual intake of nutrients and foods: a review of the theory. J Am Diet Assoc.

